# KF-Containing Interphase Formation Enables Better Potassium Ion Storage Capability

**DOI:** 10.3390/molecules29132996

**Published:** 2024-06-24

**Authors:** Tianyi Zhang, Ning Yuan, Zijie Li, Kun Chao, Zhonghua Zhang, Guicun Li

**Affiliations:** 1 College of Materials Science and Engineering, Qingdao University of Science and Technology, Qingdao 266042, China; 2 College of Electromechanical Engineering, Qingdao University of Science and Technology, Qingdao 266061, China

**Keywords:** potassium ion batteries, bismuth anodes, electrolytes, nanostructures, nanocomposites

## Abstract

Rechargeable potassium ion batteries have long been regarded as one alternative to conventional lithium ion batteries because of their resource sustainability and cost advantages. However, the compatibility between anodes and electrolytes remains to be resolved, impeding their commercial adoption. In this work, the K-ion storage properties of Bi nanoparticles encapsulated in N-doped carbon nanocomposites have been examined in two typical electrolyte solutions, which show a significant effect on potassium insertion/removal processes. In a KFSI-based electrolyte, the N-C@Bi nanocomposites exhibit a high specific capacity of 255.2 mAh g^−1^ at 0.5 A g^−1^, which remains at 245.6 mAh g^−1^ after 50 cycles, corresponding to a high capacity retention rate of 96.24%. In a KPF_6_-based electrolyte, the N-C@Bi nanocomposites show a specific capacity of 209.0 mAh g^−1^, which remains at 71.5 mAh g^−1^ after 50 cycles, corresponding to an inferior capacity retention rate of only 34.21%. Post-investigations reveal the formation of a KF interphase derived from salt decomposition and an intact rod-like morphology after cycling in K2 electrolytes, which are responsible for better K-ion storage properties.

## 1. Introduction

Rechargeable potassium ion batteries (KIBs) have attracted increasing attention due to the high natural abundance of K elements and suitable redox potential (−2.93 V vs. SHE.) [1,2,3,4,5]. In addition, the lower cost of salts for K-ion electrolytes and lower de-solvation kinetic barriers also make KIBs a commercially feasible alternative to conventional lithium ion battery technologies [6,7,8]. Specifically, KIBs are composed of cathodes, anodes and K-ion electrolytes. The compatibility between anodes and K-ion electrolytes holds the key to the stability of cycles of KIBs, which still remains a challenge [9,10,11].

Typically, graphite can serve as an anode for KIBs, which display a specific capacity of 279 mAh g^−1^ and a relatively low potential of 0.1 V vs. K^+^/K. However, graphite anodes encounter the great challenges of limited capacity and the potential dendrite growth of K-metal because of their low working potential [12,13,14]. Interestingly, bismuth, with alloying and de-alloying working mechanisms, has been considered as one of the most promising alternatives to graphite anodes due to its larger specific capacity (384 mAh g^−1^) and more suitable operating voltage range (voltage plateau of 0.25 V) [15,16,17]. Nevertheless, a significant challenge hindering its commercialization still exists, which is the inevitable volume expansion (approximately 406%) during repeated potassium insertion/removal processes, which can lead to structural instability and rapid capacity decay [18,19,20].

Confining Bi nanoparticles into a highly conductive and porous carbon skeleton represents an efficient solution to mitigate its volume expansion and capacity degradation [21,22,23]. Yin and co-workers have reported Bi nanoparticles encapsulated in mesoporous carbon nanofibers through the electrospinning method, which displayed a reversible capacity of 163.3 mAh g^−1^ at relatively high rate [24]. Xu and co-workers have presented carbon-encapsulated Bi/Bi_2_O_3_ heterostructures for KIBs, which demonstrated good capacity retention after 350 cycles (251.8 mAh g^−1^) [23]. A composite of Bi@Bi_2_O_3_ anchored on porous graphene has also been reported to show good capacity retention after 100 cycles [25].

Despite these achievements, the performances of Bi-based compound anodes still need to be improved. In addition, the charge and discharge behavior of Bi-based anodes in varied electrolyte solutions is rarely investigated, which may have a potential impact on the electrode and electrolyte interphases and, consequently, the K-ion storage properties.

In this work, Bi nanoparticles confined by N-doped carbon (abbreviated as “N-C@Bi”) nanocomposites have been prepared based on a Bi-containing metal organic framework (MOF), and their K-ion storage properties have also been examined with two electrolyte solutions. The as-prepared N-C@Bi nanocomposites display rod-like morphology, finely distributed Bi nanoparticles with a diameter of ~18 nm, and abundant N/O-doped sites. In 0.8 M KPF_6_/EC+DEC electrolyte (K1), the N-C@Bi nanocomposites show a specific capacity of 209.0 mAh g^−1^, which remains at 71.5 mAh g^−1^ after 50 cycles, corresponding to an inferior capacity retention rate of only 34.21%. In 3.0 M KFSI/DME (K2) electrolyte, the N-C@Bi nanocomposites exhibit a high specific capacity of 255.2 mAh g^−1^ at 0.5 A g^−1^, which remains at 245.6 mAh g^−1^ after 50 cycles, corresponding to a high capacity retention rate of 96.24%. Post-investigations reveal the formation of a KF interphase derived from salt decomposition and the intact rod-like morphology after cycling in the K2 electrolyte, which might be responsible for better K-ion storage properties. This work highlights the importance of electrolyte solutions for alloying-type anodes for batteries applications.

## 2. Results and Discussions

**Figure 1:**
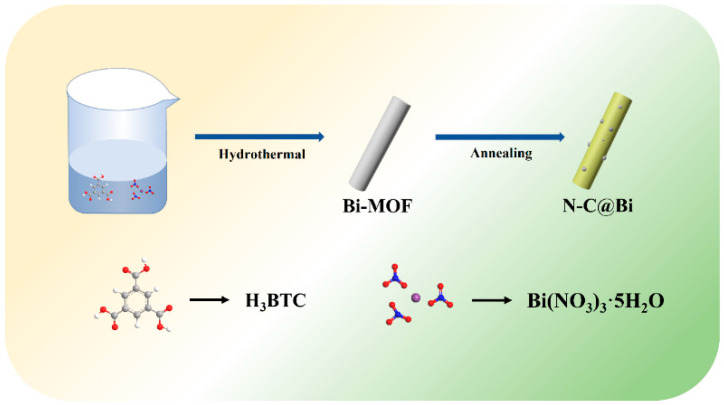
Schematic illustration of synthesis procedure of the N-C@Bi nanocomposites.

The synthesis process is shown in Figure 1. Firstly, 1,3,5 benzoic acid and bismuth nitrate pentahydrate are dispersed in 60 mL of methanol, followed by a solvothermal reaction to obtain white Bi-MOF. The unreacted Bi^+^ is removed by centrifugation and washing. Subsequently, the sample is dried and heated to 700 °C in an argon atmosphere in a tube furnace, and kept for 4 h, and then, N-C@Bi nanoparticles are obtained.

**Figure 2:**
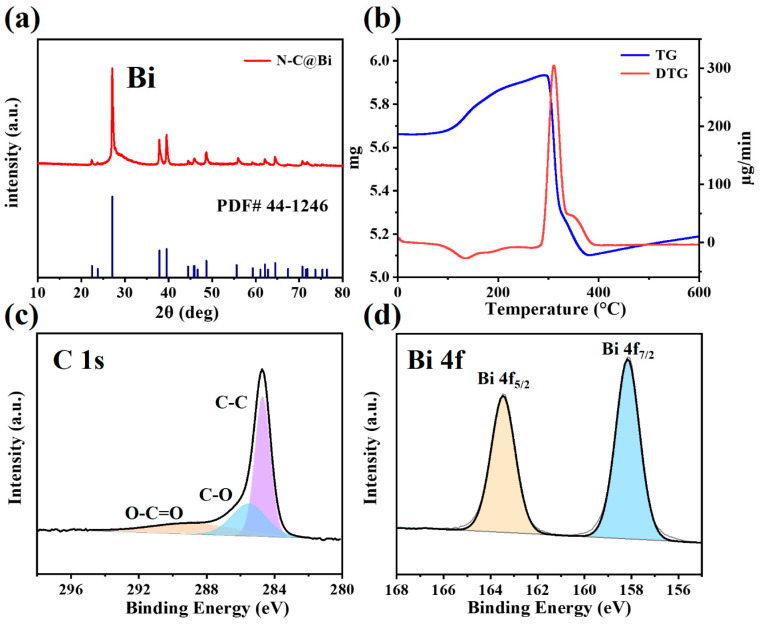
XRD pattern (**a**) and TG curve (**b**) of N-C@Bi nanocomposites (Blue line in a shows the standard XRD pattern of Bi). C 1s (**c**) and Bi 4f (**d**) XPS spectra of N-C@Bi nanoparticles.

The crystal structure and carbon content of N-C@Bi nanocomposites were analyzed by XRD and TG. Figure 2a shows XRD patterns of N-C@Bi nanocomposites, which clearly show obvious XRD characteristic peaks at 27.17°, 37.95°, 39.17°, 48.69°, and 64.50°, corresponding to the (012), (104), (110), (202), and (122) crystal planes of Bi (PDF card: 44-1246). The main crystalline phase of nanocomposites is metallic bismuth. Figure 2b shows the TG curve of N-C@Bi nanocomposites. The weight increase in the temperature range of 100 °C to 291 °C is due to the oxidation of bismuth, during which, the oxygen element is introduced into the sample. During the subsequent heating process, a significant decrease in total mass can be observed within the temperature range of 291 °C to 382 °C. This is because the carbon and nitrogen will react with oxygen in the air to generate CO_2_ and NO*_x_*, respectively, leading to a decrease in mass. It is noted that a gradual weight increase from 400 °C to 600 °C might be also be due to the oxidation of Bi. The percentage of carbon and nitrogen in N-C@Bi nanocomposites can be calculated to be around 13% based on this mass decrease.

The surface chemical composition of the N-C@Bi nanocomposites was analyzed by XPS. As shown in Figure 2c, the C 1s XPS spectrum shows three characteristic peaks at 284.7 eV, 285.5 eV, and 288.8 eV, corresponding to the bonding states of C-C bond, C-O bond, and C-C=O bond, respectively [26]. As shown in Figure 2d, the Bi 4f XPS spectrum shows two distinct characteristic peaks at 158.2 eV and 163.5 eV, corresponding to the Bi 4f orbital bonds of bismuth elemental [23,27].

**Figure 3:**
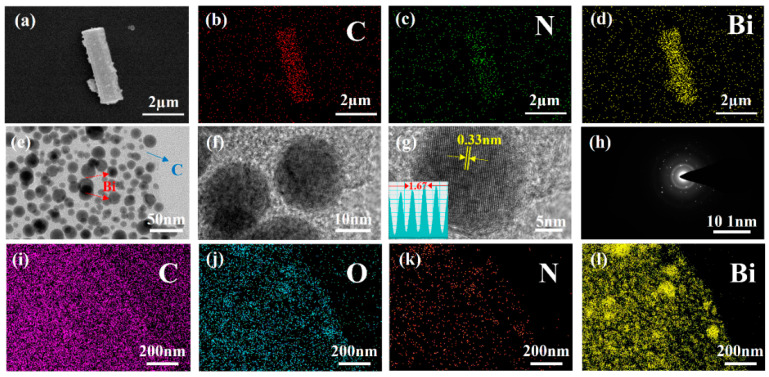
Typical SEM images and SEM-EDS (**a**–**d**) of N-C@Bi nanocomposites. TEM images (**e**), HRTEM image (**f**,**g**), SAED image (**h**), EDS-mapping image (**i**–**l**) of N-C@Bi nanocomposites.

The typical SEM image in Figure 3a reveals a cylindrical short rod with a length of about 6 µm and a diameter of about 1 µm. The surface of the sample is not smooth, and some small spherical particles can be observed, which are bismuth metal particles derived from the high-temperature reaction. The corresponding EDS-mapping images in Figure 3b–d reveal the uniform distribution of C, N, and Bi elements in the N-C@Bi nanocomposites. The TEM image in Figure 3e reveals the spherical particles of Bi, which are randomly distributed onto the carbon matrix. The diameter of the spherical particles is around 15 nm, which is conductive to ion transportation (Figure 3f). Figure 3g reveals the (012) crystal plane of bismuth metal and the carbon matrix embedding Bi nanoparticles. The good crystallinity of Bi and the amorphous nature of the carbon matrix are also reflected by the SAED image in Figure 3h. Figure 3i–l clearly show the presence of C, O, N, and Bi elements, and the allocation of oxygen is associated with bismuth, which might be due to the partial surface oxidation of Bi. The relative element ratio is listed in Table 1.

**Figure 4:**
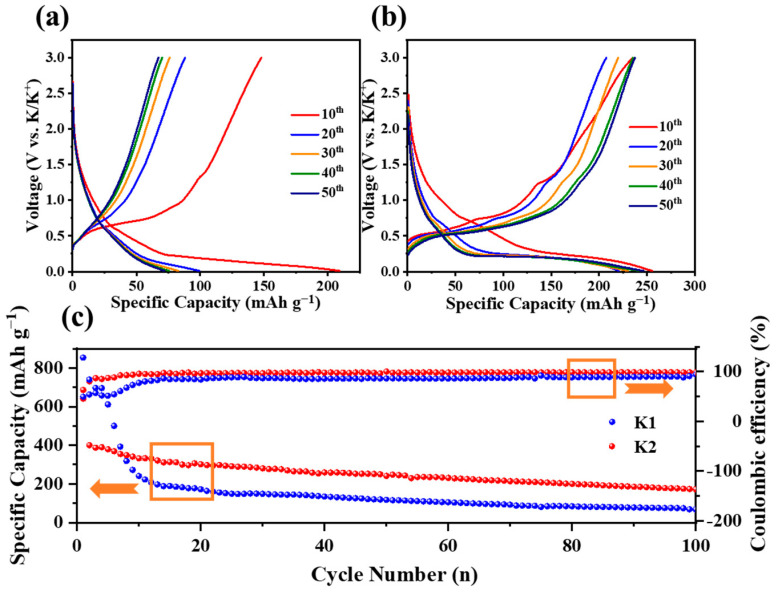
The 10th to 50th galvanostatic charge and discharge profiles of the N-C@Bi anode in the K1 electrolyte (**a**) and K2 electrolyte (**b**). (**c**) The cycle performances of the N-C@Bi anode in the K1 electrolyte and K2 electrolyte.

To verify the effect of electrolytes on the K-ion storage properties of the N-C@Bi anode, constant charge and discharge tests were conducted using K1 and K2 electrolytes at 0.5 A g^−1^. As shown in Figure 4a, the discharge capacities of the N-C@Bi anode in the K1 electrolyte are 209.0 mAh g^−1^ and 71.5 mAh g^−1^ at the 10th and 50th cycle, respectively. The capacity retention is only 34.21%. In the K2 electrolyte (Figure 4b), the discharge capacities of the N-C@Bi anode are 255.2 mAh g^−1^ and 245.6 mAh g^−1^ at the 10th and 50th cycle, respectively. The capacity retention is 96.24%, much higher than that of the K1 electrolyte. In addition, the charging and discharging voltage plateaus of the N-C@Bi anode gradually disappear in the K1 electrolyte but remain stable in the K2 electrolyte. Figure 4c shows the cycle performances of the N-C@Bi anode at 0.2 A g^−1^. During the initial cycles, the N-C@Bi anode in the K1 electrolyte shows a higher discharge capacity than that in the K2 electrolyte, which might be due to serious side electrochemical reactions with the electrolyte. This is consistent with the comparatively lower Columbic efficiencies in the K1 electrolyte, as shown in Figure 4c. It is obvious that the capacity in the K2 electrolyte (192 mAh g^−1^ after 100 cycles) is significantly higher than that in the K1 electrolyte (94 mAh g^−1^ after 100 cycles). These results demonstrate better cycling stability in the K2 electrolyte for the N-C@Bi anode.

**Figure 5:**
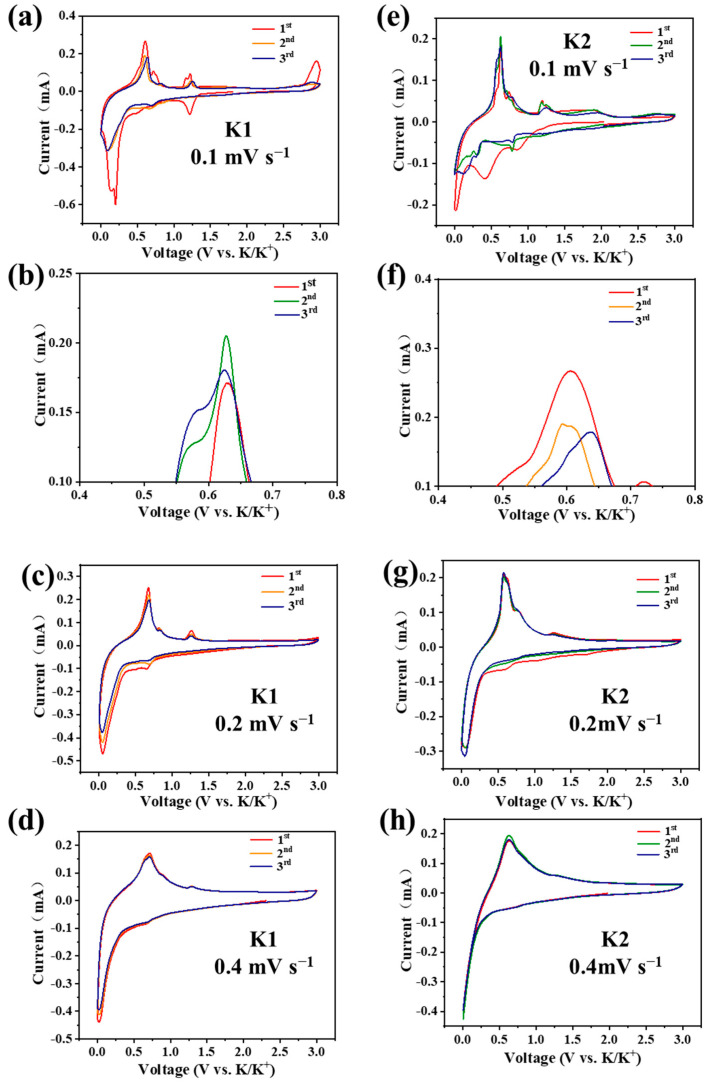
Typical CV curves of N-C@Bi anode in K1 electrolyte (**a**–**d**) and K2 electrolyte (**e**–**h**) at varying scan rates.

As shown in Figure 5a, the CV curves of the N-C@Bi anode in the K1 electrolyte have obvious reduction peaks at around 0.2 V and 0.7 V, corresponding to the two processes of potassium–bismuth alloying. There are obvious oxidation peaks at 0.61 V and 1.24 V, corresponding to the potassium–bismuth de-alloying process. It is noted that the first oxidation peak for the N-C@Bi anode in the K1 electrolyte changes from 0.61 V to 0.64 V during the first and third cycles (Figure 5b). Meanwhile, in the K2 electrolyte, the oxidation peaks are almost unchanged during the CV cycles, which are located at 0.62 V (Figure 5f). As shown in Figure 5e, the CV curves of the N-C@Bi anode in the K2 electrolyte have obvious reduction peaks at 0.2 V and 0.7 V and obvious oxidation peaks at 0.62 V and 1.24 V. At a low scan rate, the second and third CV cycles in both K1 and K2 do not overlap, which might be due to the gradual activation process during the first several cycles. Several weak peaks at lower and higher voltages can be clearly observed during the first scanning process, which might be caused by the side reaction between the electrolyte and electrode. As the scanning rates increase (Figure 5c,d,g,h), these weak peaks disappear, indicating the formation of solid–electrolyte interphases (SEIs) in both electrolytes. At a higher scan rate, the first and second CV curves of the N-C@Bi anode in the K2 electrolyte overlap better than those in the K1 electrolyte. These results suggest better cycle stability in the K2 electrolyte, which might be due to the formation of a KF-rich interphase, as discussed below.

**Figure 6:**
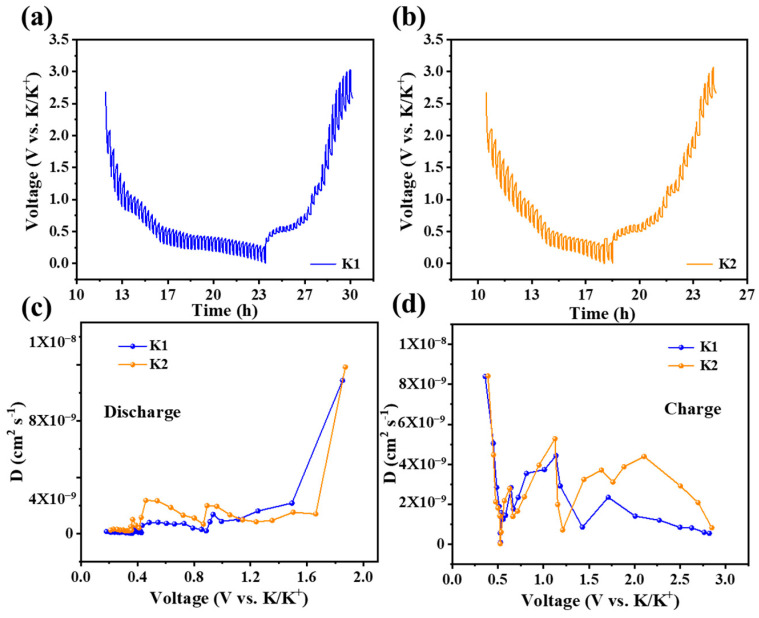
GITT curves of N-C@Bi anode in K1 electrolyte (**a**) and K2 electrolyte (**b**). K-ion diffusion coefficients of N-C@Bi electrodes in both electrolytes during discharge process (**c**) and recharge process (**d**).

To verify the reaction kinetics of the N-C@Bi electrode in two electrolytes, GITT tests were performed (Figure 6a,b). In the K2 electrolyte, the voltage difference between the working voltage and equilibrium voltage is smaller than in the K1 electrolyte, which indicates faster potassium–bismuth alloying kinetics in the former electrolyte. In addition, the value of the *D*K-ion was also calculated by Fick’s second law [28]:

(1) $$D_{K-ion}=\frac{4}{\pi \tau }{\left(\frac{m_{B}V_{M}}{M_{B}S}\right)}^{2}{\left(\frac{∆E_{S}}{∆E_{\tau }}\right)}^{2}$$

where *τ* represents the current pulse time. *m_B_* is the mass of active material in the electrode. *V_M_* is the molar volume of active material. *M_B_* is the molar mass of active material. *S* is the surface area of the electrode. Δ*E_S_* and Δ*E_τ_* are the voltage difference and time difference, respectively. As shown in Figure 6c,d, during the discharge process, the K-ion diffusion kinetics of the N-C@Bi anode in K2 electrolyte is much better than that in the K1 electrolyte within voltage range from 0.2 V to 1.1 V. During the charging process, the diffusion ability of K^+^ within the two electrolytes is basically the same, and the diffusion ability of K^+^ in the K2 electrolyte is much higher than K1 electrolytes at a high voltage. Faster K-ion kinetics in the K2 electrolyte might be responsible for the lower overpotential and higher specific capacity for the N-C@Bi electrode.

**Figure 7:**
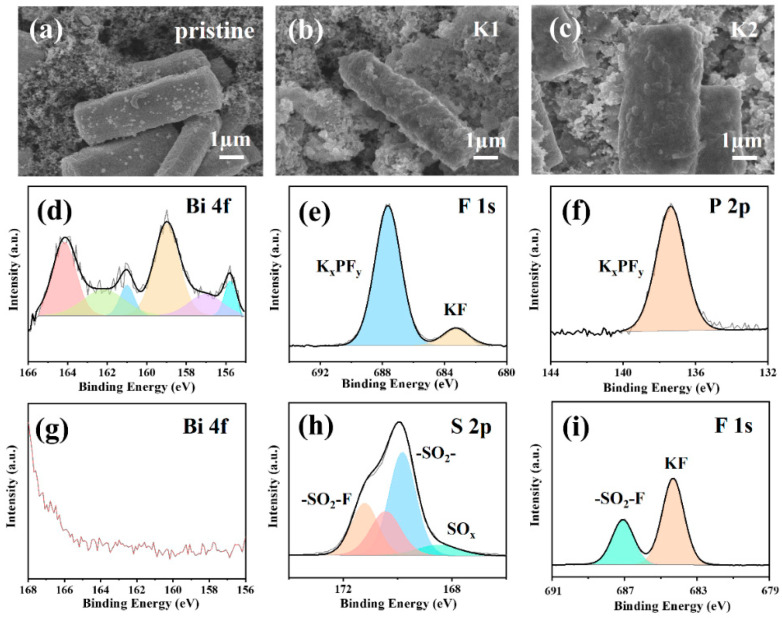
SEM images of pristine N-C@Bi anode (**a**) and cycled N-C@Bi anode in K1 electrolyte (**b**) and K2 electrolyte (**c**). Bi 4f (**d**), F 2p (**e**), and P 2p (**f**) XPS spectrum of cycled N-C@Bi anode in K1 electrolyte. Bi 4f (**g**), S 2p (**h**), and F 1s (**i**) XPS spectrum of cycled N-C@Bi anode in K2 electrolyte.

The electrolyte solutions are shown to have a significant influence on the K-ion storage ability, which might be due to the decomposition of salt and solvents. To prove this conjecture, SEM images of a cycled N-C@Bi anode with varying electrolytes have been characterized, as shown in Figure 7a–c. The pristine N-C@Bi anode shows a similar morphology to the N-C@Bi nanocomposites. Fine Super-P particles are also observed which surround the N-C@Bi nanocomposites (Figure 7a). After cycling in the K1 electrolyte, the surface of the N-C@Bi anode changes significantly. An uneven and unsmooth electrode surface is observed, which might be caused by the formation of an uneven and thick SEI layer. As shown in Figure 7c, SEM images of the cycled N-C@Bi anode in the K2 electrolyte show that its surface remains smooth and flat. This indicates that a thin and uniform SEI layer is generated on the surface of the cycled N-C@Bi anode in the K2 electrolyte.

To check the composition of the SEI layer, XPS tests have been conducted. The characteristic peaks in Bi *4f* XPS spectra can be clearly observed, which reveal the existence of Bi^0^ and Bi^3+^ species [23]. Since the main component of the K1 electrolyte is KPF_6_, the characteristic peaks of K_x_PF_y_ and KF can be clearly observed in the F *1s* XPS spectra [1,29]. These phosphides are also the main constituents of the inorganic SEI layer. A distinct characteristic peak can be observed in the P *2p* XPS spectra, indicating that its P element is mainly present in the K_x_PF_y_ form [29]. As for the K2 electrolyte, no characteristic peak of Bi *4f* XPS spectra is observed (Figure 7g). This might indicate that the SEI layer formed after cycling in the K2 electrolyte is a dense protective layer, which can completely wrap the N-C@Bi anode and prevent it from side reactions with electrolytes. In the S *2p* XPS spectra (Figure 7h), clear characteristic peaks of -SO_2_-F, -SO_2_-, and SO_x_ can be observed [30]. In F *1s* XPS spectra, characteristic peaks of SO_2_-F and KF can be clearly observed, and the peak of KF is more obvious [1,29]. This suggests the formation of a KF-containing SEI layer in the K2 electrolyte, which can effectively protect the N-C@Bi anode from degradation during repeated cycling.

## 3. Experimental Section

### 3.1. Materials Syntheses

The synthesis of Bi-MOF was analogous to previous reports [27,31,32]. Typically, 768.90 mg of trimesic acid (H_3_BTC) (>98%) and 149.88 mg of Bi(NO_3_)_3_·5H_2_O (>99%) were dispersed into 60 mL of methanol at room temperature. After continuous stirring, it was transferred into a Teflon-lined steel reactor (100 mL in volume). The solvothermal reaction was maintained at 120 °C for 24 h. Finally, all the resultant products were centrifuged and washed three times with methanol in sequence, which were dried at 60 °C under vacuum for 12 h to obtain the Bi-containing MOF (Bi-MOF) precursor.

Bi-MOF was placed in a porcelain boat. Then, a porcelain boat was placed in a tube furnace and heated at 700 °C (heating rate is around 5 °C min^−1^) for 4 h to obtain the N-C@Bi nanocomposites.

### 3.2. Materials Characterizations

The microstructures of the samples were characterized by typical scanning electron microscope (SEM, JSM 6700F, Japan Electronics Co., Ltd., Tokyo, Japan), energy dispersive spectroscope (EDS, JEOL-6300F), and transmission electron microscope (TEM, JEM-2100 Plus, Japan Electronics Co., Ltd., Tokyo, Japan) measurements. (The working voltage was 10 KV, and the current was 8 μA.) Powder X-ray diffraction (XRD, Rigaku Smartlab, Rigaku Corporation of Japan, Osaka City, Japan) was used to characterize the crystal structures of the samples with Cu K_α_ radiation (λ = 1.5406 Å), and the diffraction angle ranged from 10° to 80°. X-ray photoelectron spectroscopy (XPS) measurements were tested on an AXIS Ultra DLD electron spectrometer. The X-ray source was Al Ka (1486.6 eV) (data presentation was achieved through Origin2021 and MDI JADE 6 software). The thermal stability and carbon content of the sample were analyzed by thermogravimetric (TG) tests. The sample was heated in air at 10 °C min^−1^, and the temperature range was from 20 °C to 800 °C. (Data fitting was achieved through Origin2021 software).

### 3.3. Electrode Preparation, Battery Assembly and Tests

The anodes were prepared by mixing the N-C@Bi nanocomposites, Super-P, and polyvinylidene fluoride with 7:2:1 in N-methyl-2-pyrrolidone (NMP) solvent. The mixed slurry was evenly coated on a copper foil, and then, it was put into a vacuum drying oven and dried under 120 °C for 12 h. The anodes were shaped into a circular pellet which was 12 mm in diameter and had a mass loading of ~0.6 mg cm^−2^. The CR2032-type coin cells were assembled in an Ar-filled glovebox with O_2_ and H_2_O concentrations of <0.01 ppm. Potassium metal was used as the counter electrode, and glass fiber was selected as a separator. The adopted electrolyte was prepared by mixing KPF_6_ (0.8 M) into ethylene carbonate and diethyl carbonate (1:1) (namely 0.8 M KPF_6_/EC + DEC electrolyte, abbreviated as the “K1” electrolyte) and KFSI (3.0 M) into ethylene glycol dimethyl ether (namely 3.0 M KFSI/DME, abbreviated as the “K2” electrolyte).

The cycle performance and galvanostatic intermittent titration technique (GITT) of the N-C@Bi anode were tested using LAND CT2001A and LAND CT3001A battery-testing systems with constant charge and discharge currents in a fixed voltage window from 0.001 to 2.0 V (data presentation was achieved through Origin and LAND V7 software). Cyclic voltammetry and electrochemical impedance spectroscopy were carried out on the AUTOLAB electrochemical workstation. CV curves were measured at different scan rates of 0.1 mV s^−1^. (Data presentation was achieved through Origin2021 and Nova 1.8.14 software).

## 4. Conclusions

In this work, N-C@Bi nanocomposites have been prepared by a facile calcination process with Bi-MOF as a template. The N-C@Bi nanocomposites serve as a good model anode for K-ion storage because they show the high crystallinity of Bi nanoparticles, highly conductive carbon nanocomposites, and abundant N/O-doping sites. In a typical electrolyte of 3.0 M KFSI/DME (K2), N-C@Bi nanocomposites exhibit superior specific capacity of 192 mAh g^−1^ after 100 cycles compared to the electrolyte of 0.8 M KPF_6_/(EC+DEC) (K1) (94 mAh g^−1^ after 100 cycles). In addition, N-C@Bi nanocomposites with the K2 electrolyte display much better K-ion diffusion kinetics and lower overpotential compared to those with the K1 electrolyte, as revealed by a series of CV and GITT tests. Post-investigations of N-C@Bi nanocomposites after cycling reveal the formation of a robust KF-containing interphase in the K2 electrolyte, which might be derived from the decomposition of KFSI salts. This work is of significance to the design of alloying-type anodes and the rational screening of electrolytes for KIBs and other battery chemistries.

## Figures and Tables

**Table 1:** Element ratio in N-C@Bi nanocomposites.

	C	N	O	Bi
Element ratio (%)	45	2	9	45

## Data Availability

Data will be made available on request.
